# What Are Children Good For?

**Published:** 1870-07

**Authors:** 


### WHAT ARE CHILDREN GOOD FOR?

BY UNCLE BOB.

Who can stop and in one short answer satisfy this question?

Does not the answer expand itself, and a multitude of replies suggest themselves?

If you say as some one but recently did to my question—that "half of them are good for nothing," I shall tell you the child is father to the man. Men are but children of a larger growth.

I sometimes want to admit that half of mankind *are* nearly worthless. We see too often, lazy, dependent men, possessing no more vitality than is necessary to a mere existence.

Keep an eye to the young father of the man. Don't let any empty rooms in his head stand unoccupied, with the doors swinging open and free, too inviting a chance for idle thoughts to enter and dwell. "Just as the twig is bent the tree is inclined," is a trueism which it is good to recall more often than once.

Let us reverse the saying with which we began—that the child is father of the man, into another equally as apt—men are but children of a larger growth. Here we have a good answer to our question—*what are children good for?*

We want to make a large growth for our men. Little dwarfish men are not the race we want to have grow up about us. Nature never expects to grow anything in its natural course beyond the capacity of the producing stalk. She preserves the great laws of supply and demand when left to her own ways. This beautiful law is illustrated constantly if we consider the vegetable world about us. So the physical life should show a like equality.

The world is calling for active, vigorous work from every one, and how unsatisfactorily are we fitting the coming generation to meet this call. Children who have been brought up by parents who forsee this good of children are not so numerous as they should be. Not only are the old fashioned family circles, as they were, numerous and hearty, becoming sadly reduced, but also the capabilities of the children are reduced.

It is useless perhaps to ask why this fault is prevailing. Some there are who don't know how to maintain a household, but the larger number, in places where society makes rules which nature never endorses, will not meet the question of the "use of children." The care and responsibility of them interferes with other occupations. Parents feel the deprivation of what are called social demands, or better called social indulgencies.

I mean, speaking in very plain terms, that this generation of parents do not like children.

Household pets are other than a bright circle of children, being educated to usefulness.

If the age of twelve or fourteen could be reached by children without the cares and trials of the preceeding eleven or thirteen, then once more might the family circle be an enlarged and happy throng. The child would not be repelled for giving an enthusiastic outburst of joy, nor be made to tremble if he dared to utter a shout before asking consent.

If a child could be born with his pants on and his hair nicely combed; if he could be introduced to the world as a perfected being with no attendant care, then would happy fathers and mothers delight to tell how large a number were theirs.

But this is not so and the other fact is so. Little families and little cares go hand in hand. The family which used to be, and which now should be, esteemed an honor, is considered a burden.

We find, too, in many of our communities an opposition to a governing restraint. The old fashioned idea of New England, that the father and mother were responsible, does not prevail.

Young heads who are not capable of directing the hands to dress or undressing the bodies who support them, are allowed to select their own articles of food and style of amusement.

If the absurdity of these indulgences could be brought to the attentive notice of parents more frequently, the successful sale of soothing syrups and salves would be reduced. It is ridiculous to conceive that a child should exercise the judgment which the parents have not fully learned. Ridiculous that the tears of a child should be allowed to win a coveted thing which has been refused.

So children are spoiled, and so households which should be happy in health, are made burdensome by sickness and cares.

That children are good for anything is the wonder to many, and that anything is good for children is the belief of too many.

